# Reduced-portion entrées in a worksite and restaurant setting: impact on food consumption and waste

**DOI:** 10.1017/S1368980016001348

**Published:** 2016-06-03

**Authors:** Sarah Berkowitz, Len Marquart, Elton Mykerezi, Dennis Degeneffe, Marla Reicks

**Affiliations:** 1 Department of Food Science and Nutrition, University of Minnesota, 1334 Eckles Avenue, St. Paul, MN 55108, USA; 2 Department of Applied Economics, University of Minnesota, St. Paul, MN, USA; 3 Consumer Centric Solutions LLC, St. Paul, MN, USA

**Keywords:** Reduced-size entrées, Worksite cafeteria, Restaurant, Adults, Food service

## Abstract

**Objective:**

Large portion sizes in restaurants have been identified as a public health risk. The purpose of the present study was to determine whether customers in two different food-service operator segments (non-commercial worksite cafeteria and commercial upscale restaurant) would select reduced-portion menu items and the impact of selecting reduced-portion menu items on energy and nutrient intakes and plate waste.

**Design:**

Consumption and plate waste data were collected for 5 weeks before and 7 weeks after introduction of five reduced-size entrées in a worksite lunch cafeteria and for 3 weeks before and 4 weeks after introduction of five reduced-size dinner entrées in a restaurant setting. Full-size entrées were available throughout the entire study periods.

**Setting:**

A worksite cafeteria and a commercial upscale restaurant in a large US Midwestern metropolitan area.

**Subjects:**

Adult worksite employees and restaurant patrons.

**Results:**

Reduced-size entrées accounted for 5·3–12·8 % and 18·8–31·3 % of total entrées selected in the worksite and restaurant settings, respectively. Food waste, energy intake and intakes of total fat, saturated fat, cholesterol, Na, fibre, Ca, K and Fe were significantly lower when both full- and reduced-size entrées were served in the worksite setting and in the restaurant setting compared with when only full-size entrées were served.

**Conclusions:**

A relatively small proportion of reduced-size entrées were selected but still resulted in reductions in overall energy and nutrient intakes. These outcomes could serve as the foundation for future studies to determine strategies to enhance acceptance of reduced-portion menu items in restaurant settings.

Food prepared away from home accounts for a high proportion of energy consumed and money spent on food in the USA. Nationally representative dietary intake data collected between 2005 and 2008 showed that about 32 % of energy consumed was from away-from-home foods^(^ ^1^ ^)^. More recently, away-from-home foods were shown to account for about 43 % of total food expenditures for all families and individuals in 2013^(^ ^2^ ^)^. These findings are of concern because a number of studies have shown that away-from-home foods contribute to higher energy intake, reduced diet quality, and increased risk of obesity and biomarkers of chronic disease in adults^(^ ^1^ ^–^ ^7^ ^)^. In general, away-from-home foods are high in energy, saturated fat and Na^(^ ^1^ ^,^ ^4^ ^,^ ^6^ ^,^ ^7^ ^)^.

The high energy content of away-from-home foods has been correlated with large portion sizes^(^ ^8^ ^)^, which have increased in the USA since the 1970s^(^ ^9^ ^–^ ^11^ ^)^. The number of large-sized portions introduced by restaurants from 1960 to 2009 increased in a parallel manner to energy in the US food supply^(^ ^9^ ^)^. These trends resulted in a call for action for restaurant owners to reduce portion size or offer some proportion of their offerings in reduced sizes^(^ ^9^ ^)^.

Studies that have examined the impact of adding reduced-size entrées to the menu on consumer willingness to purchase reduced-size entrées in food-service establishments are limited, with variable methodology between studies. Vermeer *et al*.^(^ ^12^ ^)^ introduced small portion sizes (roughly two-thirds of existing size) in addition to the regular-sized portions in seventeen workplace cafeterias in the Netherlands with either proportional or value-size pricing. An additional eight cafeterias served as controls. The proportion of reduced-size entrée sales in relation to the regular-size entrée sales was approximately 10 % regardless of pricing condition. Reduced-size entrées were purchased most often by women. Half-size portions were offered in a Midwestern US restaurant in addition to the regular-sized portions for a 2-month period^(^ ^13^ ^,^ ^14^ ^)^. The average monthly unit sales of the regular-sized portions decreased by 41 % (297 meals), but the average sales of half-size portions increased from 0 to 949 units per month. Schwartz *et al*.^(^ ^15^ ^)^ offered university students the option of downsizing side dishes at a Chinese restaurant on campus and found that about one-third were willing to downsize the dish when asked explicitly.

Several studies that have examined the effects of decreasing or increasing portion sizes on food intake by customers in food-service establishments have used plate waste methods to estimate food intake^(^ ^16^ ^,^ ^17^ ^)^. For example, in a university dining facility, the portion size of French fries was decreased by about 15 g/week over a 3-week period while researchers monitored the production of French fries and collected uneaten French fries at the tray return area^(^ ^16^ ^)^. In another study conducted in a university cafeteria-style restaurant, each dish containing a standard and large portion entrée was weighed in the kitchen before the meal^(^ ^17^ ^)^. After the meal, plate waste (uneaten portion of each entrée) was weighed in the kitchen to determine the portion consumed.

A large number of studies on portion size manipulation and effects on food intake have focused on the ‘portion size effect’ where offering larger portions leads to greater intake^(^ ^18^ ^)^, whereas limited studies have examined consumer willingness to select smaller portion-sized menu options^(^ ^12^ ^–^ ^15^ ^)^. Studies reporting the impact of reduced-size options have frequently focused on energy intake^(^ ^15^ ^,^ ^16^ ^)^ but not on nutrient intakes. Therefore, the objective of the present study was to determine selection rates of reduced-size entrées offered in worksite cafeteria lunch meals and restaurant dinner meals and the impact on energy and nutrient intakes and plate waste. The study was designed to test the hypothesis that offering reduced- and full-size entrées would result in decreased energy and nutrient consumption and plate waste compared with offering only full-size entrées.

## Methods

### Participants

The current study was conducted in two food-service establishments in the Minneapolis/St. Paul metropolitan area, Minnesota, USA, from April to July 2013. The sites were identified based on recommendations of a food-service research consultancy and through personal contacts. In one setting, a worksite cafeteria (non-commercial setting) served lunch to about 125–200 employees daily. In the other setting, a private golf club restaurant (commercial upscale white tablecloth) served dinner to thirty to fifty patrons on weekdays and fifty to seventy-five patrons on weekends. Over 500 members paid an annual fee to join, with $US 250 credited to an account quarterly to cover dining expenses. The University of Minnesota Institutional Review Board reviewed the study and confirmed that the criteria for exempt status were met given that the data were based on observation of public behaviour.

### Study procedures

At the worksite cafeteria, employees could choose from the entrée of the day, cold and hot sandwiches, soup or salad bar for their lunch meal. The entrée, full sandwiches and a large salad from the salad bar were priced at $US 3·00 per meal. A small salad from the salad bar, a bowl of soup and half a sandwich were priced at $US 1·50 each. Employees could pay by cash or pre-purchase discounted meal tickets worth $US 3·00 each. Food costs were subsidized by the employer as an employee benefit; therefore the cafeteria could charge lower prices for lunch meals and expect high participation from employees.

Plate waste and consumption data were collected for 12 weeks, including 5 weeks prior to the introduction of reduced-size entrées (baseline period) and for the next 7 weeks when both reduced-size and full-size entrées were offered (intervention period) for each entrée of the day. Reduced-size entrées were priced at $US 1·50. For the intervention period, a sign informing employees that reduced-size entrées were available and a plated reduced-size entrée to display the difference in size compared with full-size entrées were placed at the hot food station. Selection data were collected for all days within the 12-week study period. Due to menu changes that occurred throughout the study, data from six entrées were available to compare consumption and plate waste data for the baseline and intervention periods (meatloaf, spaghetti, pork loin, lasagne, chopped steak and chicken parmesan).

At the restaurant, each month the chef created a new menu including a number of appetizers and desserts and between six and eight entrées such as steaks, fish and pasta, which ranged in price from $US 18 to $US 32. Additionally, a monthly bar menu was created, which included small plates and appetizers, and ranged in price from $US 7 to $US 15. Each time members dined, they received both menus as well as a list of two or three daily specials. Prior to the intervention period, reduced-size portions existed for a single entrée. The filet mignon had been available in 113 g, 170 g and 227 g (4 oz, 6 oz and 8 oz) serving sizes for one year prior to the current study.

Plate waste and consumption data were collected for dinner meals on Thursdays, Fridays and Saturdays for 7 weeks, including 3 weeks prior to the introduction of five additional reduced-size entrées and one appetizer (baseline period) and for the next 4 weeks when both reduced-size and full-size entrées were offered (intervention period). The additional reduced-size entrées included strip steak, halibut, walleye, salmon and lamb chops. Each entrée included a vegetable such as asparagus, carrot batons, garlic spinach, smashed peas or spring vegetables and a starch such as roasted new potatoes, spring vegetable risotto or caramelized onion mashed potatoes. The newly added reduced-size entrées were priced at $US 15–17. This enabled the operator to maintain approximately the same item profit margin for the reduced item as the full-sized portion, so direct substitutions would have a neutral or positive affect on profitability, although financial data were not made available for analysis. All entrées with the exception of the vegetarian option were available in full and reduced sizes during the intervention period. Selection data were collected for all days within the 7-week period.

In the worksite cafeteria and restaurant settings, several strategies were used to facilitate selection of the reduced-size entrées as suggested by Riis^(^ ^19^ ^)^ based on marketing and behavioural economics principles. These included offering more small-size entrées and increasing the visibility of reduced-size portions by providing information about their availability using signage or including as menu options, and by placing a sample reduced-size entrée on the cafeteria line.

### Plate waste data collection

Plate waste was collected and weighed by researchers at the tray disposal area in the worksite cafeteria. Plate waste was collected from servers who returned plates to the dishwashing area in the restaurant and weighed by researchers. In both settings, waste was recorded for the entrée and side dishes separately, and for the whole plate. The amount consumed by each patron was determined by subtracting the plate waste of a particular item from the average serving weight.

Recipes were obtained from the manager of the worksite cafeteria and the restaurant chef. The recipes were entered into the Nutrition Data System for Research, NDSR (University of Minnesota, Nutrition Coordinating Center ©2013). The energy and nutrient contents (total fat, saturated fat, cholesterol, Na, Ca, fibre and K) of the served portion were determined. Analysis was conducted for each plated menu item based on mean serving weight, calculated from fifteen sample weights of each menu item. Energy content for the full-size entrées served at the worksite cafeteria ranged from 2305 kJ (551 kcal) for lasagne to 2958 kJ (707 kcal) for meatloaf. Energy content for the full-size entrées served at the restaurant ranged from 2561 kJ (612 kcal) for salmon to 5477 kJ (1309 kcal) for lamb chops. The reduced-size entrées typically provided about half of the energy and nutrient content of the full-size entrées.

### Data analysis

A two proportions *z* test showed that a relatively equal number of customers purchased each of five entrées in the worksite cafeteria (meatloaf, spaghetti, pork loin, lasagne, chopped steak) when only the full-size entrées were offered during the baseline period (56–71 %) and when both the full- and reduced-size entrées were offered during the intervention period (62–65 %). These proportions were not equal for chicken parmesan (*P*=0·028); therefore energy and nutrient analyses were not completed for this entrée.

Differences in energy and nutrient intakes and food waste in the worksite cafeteria and restaurant settings for the baseline period compared with the intervention period were determined using *t* tests. All data were analysed using the statistical software package SAS version 9·3 with the significance level set at *P*<0·05.

## Results

In the worksite cafeteria, energy and nutrient intakes were significantly lower during the intervention period compared with the baseline period (Table 1). The energy consumed decreased from a mean of 2632 kJ (629 kcal) when only the full-size entrées were offered to 2322 kJ (555 kcal) when both the full- and reduced-size entrées were offered (*P*<0·0001). Nutrients of concern, including total fat, saturated fat, cholesterol and Na, were also significantly lower when both the full- and reduced-size entrées were offered (*P*<0·0001). In addition to the decreased intakes of nutrients of concern, decreased intakes of shortfall nutrients, such as fibre, Ca and K, were also observed (*P*<0·0005) during the intervention compared with the baseline period. A significant decrease in plate waste was also observed when both the full- and reduced-size entrées were offered (29·7 g) during the intervention period compared with when only the full-size entrées (45·5 g) were offered (*P*<0·0001) during the baseline period.

**Table 1:** Energy and nutrient intakes for baseline *v*. intervention periods in the worksite cafeteria and restaurant settings, Minneapolis/St. Paul metropolitan area, Minnesota, USA, April–July 2013

	Worksite cafeteria				Restaurant				
	Baseline period* (*n* 521)		Intervention period* (*n* 603)		Baseline period* (*n* 90)		Intervention period* (*n* 95)		
Energy and nutrients	Mean	se	Mean	se	Mean	se	Mean	se	*P* value†
Energy (kJ)	2632	18	2322	22	2908	102	1644	60	<0·0001
Energy (kcal)	629	4·4	555	5·2	695	24·4	393	14·3	<0·0001
Total fat (g)	34·0	0·4	29·7	0·4	40·3	2·1	21·8	0·9	<0·0001
Saturated fat (g)	16·1	0·2	13·9	0·2	18·4	0·9	10·9	0·5	<0·0001
Cholesterol (mg)	141	2·4	122	2·3	187	9·3	108	4·7	<0·0001
Na (mg)	955	13	849	13	1396	43	934	42	<0·0001
Fibre (g)	4·6	0·1	4·1	0·7	4·4	0·2	2·6	0·1	<0·0001
Ca (mg)	291	8·4	238	6·1	168	11·3	82	5·5	<0·0001
K (mg)	796	13·8	728	13·4	1397	38·6	863	35·4	<0·0001

* Baseline period, only full-size entrées offered; intervention period, both full- and reduced-size entrées offered.

† *P* value for difference between baseline and intervention periods according to *t* test (*P*<0·05).

Selection of reduced-size entrées was lowest (5·3 % of total entrées selected) during the first week they were offered, whereas selection was greater than 10 % of total entrées selected in several subsequent weeks (Table 2). The cafeteria manager indicated that some cafeteria patrons were confused regarding the use of pre-paid meal tickets to pay for reduced-size entrées during the first week they were offered. Selection of reduced-size entrées increased during the next 5 weeks, ranging from 8·2 to 12·8 % of total weekly entrées selected.

**Table 2:** Selection of reduced-size entrées by week* in the worksite cafeteria and restaurant settings, Minneapolis/St. Paul metropolitan area, Minnesota, USA, April–July 2013

	Total entrées selected (*n*)	Reduced-size entrées selected (*n*)	%
Worksite cafeteria			
Introduction of reduced-size entrées			
Week 1	569	30	5·3
Week 2	519	60	11·6
Week 3	633	81	12·8
Week 4	436	36	8·3
Week 5	746	61	8·2
Week 6	603	65	10·8
Restaurant			
Time relative to menu change			
3 weeks before†	84	5	6·0
2 weeks before†	75	10	13·3
1 week before†	111	12	10·8
Week of menu change	83	26	31·3
1st week after	112	21	18·8
2nd week after	123	32	26·0
3rd week after	109	32	29·4

* Values were derived from cafeteria selection reports and observed choice data from the restaurant.

† One menu item was offered in two reduced sizes prior to the introduction of additional reduced-size entrées (filet mignon at 113 or 170 g *v*. 227 g (4 or 6 oz *v*. 8 oz) portion).

In the restaurant setting, energy intake was reduced significantly when reduced-size entrées and full-size entrées were offered (intervention period) compared with when only full-size entrées were offered (baseline period; Table 1). Energy consumed decreased from a mean of 2908 kJ (695 kcal) to 1644 kJ (393 kcal; *P*<0·0001). Intakes of all nutrients also decreased significantly. Plate waste was reduced during the intervention period (45 g (1·6 oz)/plate) compared with the baseline period (77 g (2·7 oz)/plate; *P*<0·0051).

During the period prior to introducing the reduced-size entrées in the restaurant, the filet mignon entrée was available in multiple portion sizes: 113, 170 and 227 g (4, 6 and 8 oz). For the 3 weeks prior to offering additional reduced-size entrées, selection of the 113 and 170 g (4 and 6 oz) filet mignon accounted for between 6·0 and 13·3 % of total weekly entrées selected, respectively (Table 2). The introduction of the five additional reduced-size entrées increased the percentage of total entrées selected attributable to reduced-size entrées to 31·3 % in the week of introduction, 18·8 % in the second week post introduction, and 26·0 and 29·4 % in the following weeks (Table 2).

Interviews were conducted with the chef and restaurant manager and the cafeteria manager after the study was completed. The cafeteria manager indicated that the addition of reduced-size portions was successful for selected entrées because less food was prepared, thus reducing costs. Customer feedback indicated that they had greater flexibility to reduce intake or to pair a reduced-size entrée with other menu items, such as a side salad or a bowl of soup. The cafeteria continued to offer reduced-size entrées for selected entrées after the completion of the study. The chef and restaurant manager also indicated that the addition of reduced-size entrées to their menu was successful. According to the chef, ‘We are making 55–60 % of the price of a full portion … and when you sell items at a higher margin, you can’t lose on that.’ The restaurant’s dining committee, made up of club members, asked the restaurant to continue offering reduced-size entrées. The chef indicated that the reduced-size entrées appealed to two different segments of their membership, older and younger members, who ate at the club multiple times per week. Some initial challenges were addressed through additional staff training as servers became accustomed to offering and serving reduced-size entrées.

## Discussion

### Selection

Selection of reduced-size entrées in both settings accounted for a small proportion of total entrées selected (10–26 %), similar to findings by Vermeer *et al*.^(^ ^12^ ^)^ in worksite cafeterias in the Netherlands. In the restaurant setting in the current study, selection of reduced-size entrées increased from an average of 10 % initially to 29·4 % of total entrées selected after the additional reduced-size entrées were added to the menu. After an initial slow start in the worksite cafeteria, selection of the reduced-size entrées ranged from 8·2 to 12·8 % of total entrées selected. Factors that could explain why the selection rate varied between settings include familiarity with the concept of reduced-size portions, type of meal and cost. Because reduced-size filet mignon entrées were available in the restaurant setting prior to the introduction of other reduced-size entrées, these patrons may have been familiar with the concept of multiple portion sizes. The meal type, lunch or dinner, may have also influenced the selection of the reduced-size entrées in the two settings. Customer expectations of usual amounts to consume at lunch and dinner may have influenced selection. Lastly, the difference between the price of the full-size and reduced-size entrée may have affected selection. A saving of $US 8–9 per meal at the restaurant setting may have been more likely to motivate purchase of a reduced-size entrée at the restaurant compared with a saving of $US 1·50 at the worksite cafeteria. In the current study, prices for the reduced-size entrées were about half of the full-size entrées in both settings. This price break was possible because of the unique type of food-service setting and profit structure. For other settings, Riis^(^ ^19^ ^)^ advocated for linear pricing, with the small portion having the same unit price as the large portion, as a means of encouraging selection of small portions. However, because of the competitive environment of food-service establishments, linear pricing may have to be mandated by regulation for these establishments to adopt this practice.

### Effects on energy and nutrient intakes

When reduced-size and full-size entrées were offered in the current study, intakes of fat, saturated fat, cholesterol and Na were lower compared with when only full-size entrées were offered. Since most chain-restaurant entrées exceed US Department of Agriculture guidelines for energy, total fat, Na and saturated fat^(^ ^4^ ^,^ ^20^ ^)^, reducing portion sizes may help consumers meet the Department’s recommendations for energy intake and intakes of nutrients of concern. These nutrients include fat, saturated fat and Na, whose excess consumption is associated with obesity, hypertension, heart disease, diabetes and cancer^(^ ^21^ ^–^ ^23^ ^)^. Benefits from these reductions may be offset somewhat by the parallel decrease in intake of common shortfall nutrients such as fibre, Ca and K. Based on nationally representative dietary intake data, foods consumed at home included more fruit, dairy and whole grains compared with foods consumed away from home^(^ ^23^ ^)^. Reducing the portion size of the meal does not alter the original nutrient density of the meal, so reducing the size of a meal already low in fruits, vegetables, dairy or whole grains will either make no change or have a negative effect. To limit any negative impact of reduced-portion sizes on shortfall nutrients and food group intakes, the amounts of fruits, vegetables, dairy and whole grains should be increased prior to down-sizing entrées.

The current study did not examine the impact of offering reduced-size entrées on selection and intake of other menu items, and consequential energy and nutrient intakes. Decreased portion size at a single meal has not been shown to increase consumption at a subsequent meal^(^ ^24^ ^)^; however, the effects of reduced-size entrées on intake of other menu items at the same meal are not well known, indicating that this may be an area for future study.

### Effects on plate waste

Reductions in plate waste were observed when reduced-size entrées were offered along with full-size entrées compared with when only full-size entrées were offered in both settings in the current study, similar to findings by Freedman and Brochado^(^ ^16^ ^)^. Reduced food waste can result in decreased food costs and waste disposal costs. This information may be helpful for owners of food-service establishments as loss of revenue is an important consideration^(^ ^25^ ^)^. Additional studies are needed to quantify the impact of reduced-size entrées on the total waste of a food-service establishment. Further research on the impact of reduced-size entrées on food waste based on different types of food-service establishments is also desirable because the tendency to waste food may vary by type of restaurant (cafeteria-style *v*. sit-down restaurant).

### Limitations

Limitations include the inability to track selection and consumption by individual participants over time due to constraints on measuring individual intake in public food-service settings. This limited the ability to determine the motivation underlying the choice of smaller-sized entrées by some individuals. The current study was also unable to determine whether patrons may have compensated for consuming fewer kilojoules at lunch or dinner during the intervention period by consuming more kilojoules at future meals or if the reduced-size entrée selection resulted in an overall decrease in daily energy intake. In the restaurant setting, researchers were unable to directly observe patrons consuming the meal; therefore consumption amounts may have been misreported if patrons shared or dropped a portion of the meal. The study was conducted in a business cafeteria setting and a country club restaurant setting, both of which have a constant client base. This may limit application of results to other types of restaurants including quick-serve and casual dining. Some patrons in the restaurant setting chose to take leftover foods home. The amount of these leftovers consumed at home could not be measured; therefore the effects of offering reduced-size entrées in these cases are not known.

## Conclusions

The present study contributes to the literature regarding the impact of reduced-size entrées on selection, energy and nutrient intakes and plate waste in two types of food-service setting. The results suggest that a relatively small portion of worksite and restaurant customers will purchase reduced-size menu items if given the opportunity. When reduced-size entrées were offered along with full-size entrées, fewer kilojoules were consumed, intakes of both nutrients of concern as well as a number of shortfall nutrients were decreased, and plate waste was decreased compared with when only full-size entrées were served.
